# Exendin‐4, a GLP‐1 receptor agonist regulates retinal capillary tone and restores microvascular patency after ischaemia–reperfusion injury

**DOI:** 10.1111/bph.15059

**Published:** 2020-05-27

**Authors:** Ruyi Zhai, Huan Xu, Fangyuan Hu, Jihong Wu, Xiangmei Kong, Xinghuai Sun

**Affiliations:** ^1^ Department of Ophthalmology and Visual Science, Eye, Ear, Nose and Throat Hospital, Shanghai Medical College Fudan University Shanghai China; ^2^ Shanghai Key Laboratory of Visual Impairment and Restoration Fudan University Shanghai China; ^3^ NHC Key Laboratory of Myopia Fudan University Shanghai China; ^4^ Laboratory of Myopia Chinese Academy of Medical Sciences Shanghai China; ^5^ State Key Laboratory of Medical Neurobiology, Institutes of Brain Science and Collaborative Innovation Center for Brain Science Fudan University Shanghai China

## Abstract

**Background and Purpose:**

The aim of this study is to investigate the vasorelaxant effect of exendin‐4, a GLP‐1 receptor agonist on retinal capillaries under normal and ischaemia–reperfusion (I/R) conditions.

**Experimental Approach:**

Capillary diameters in the whole‐mounted retina were directly observed using infrared differential interference contrast microscopy. A model of retinal I/R was established inraats,using high perfusion pressure in an anterior chamber. To assess the effects of exendin‐4, it was administered through subcutaneous injection, intravitreal injection, or eye drops. The underlying mechanism was explored by immunofluorescence, qPCR, and capillary western blots.

**Key Results:**

Immunofluorescence staining showed that GLP‐1 receptors were expressed in endothelial cells of retinal capillaries. Exendin‐4 relaxed the capillaries precontracted by noradrenaline, an effect abolished by denuding endothelium with CHAPS and inhibited by GLP‐1 receptor antagonist exendin‐9‐39, endothelial NOS (eNOS) inhibitor l‐NAME, and the guanylate cyclase blocker ODQ but not by a COX inhibitor, indomethacin. Retinal capillaries were constricted in I/R injury, an effect reversed by perfusion of exendin‐4. Expression of PI3K and Akt, phosphorylation level of eNOS and NO production after I/R were lower than that in the normal control group. Administration of exendin‐4 improved the changes.

**Conclusion and Implications:**

Exendin‐4 can restore injured microvascular patency in I/R. Exendin‐4 may regulate retinal capillaries through the GLP‐1 receptor‐PI3K/Akt‐eNOS/NO‐cGMP pathway. Therefore, exendin‐4 may be an effective treatment for improving tissue perfusion in I/R‐related conditions.

## Graphical abstract

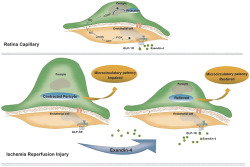

**Abbreviations**
EGM: endothelial growth medium
eNOS: endothelium NOS
GLP‐1: glucagon‐like peptide‐1
HRMEC: human microvascular endothelial cell
HRP: human retinal pericytes
I/R: ischaemia–reperfusion
ODQ: 1*H*‐[1,2,4]oxadiazolo[4,3‐*a*]quinoxalin‐1‐one
OGD/R: oxygen‐glucose deprived with reperfusion

**What is already known**

- Ischaemia–reperfusion can impair microvascular flow and thus negatively affect tissue survival.

**What does this study add**

- The GLP‐1 receptor agonist, exendin‐4, restored capillary patency following ischaemia–reperfusion injury.

**What is the clinical significance**

- Exendin‐4 may be an effective treatment for improving tissue perfusion in I/R‐related conditions.

## 1 INTRODUCTION

Exendin‐4, an agonist of GLP‐1 receptors, is widely used under the trade name Exenatide in the clinical treatment of Type 2 diabetes. More studies are showing that GLP‐1 receptor agonists, in addition to having a hypoglycaemic effect, may reduce the incidence of adverse cardiovascular events (Marso et al., 2016; Marso et al., 2016). As a result, such agonists have been recommended by the American Diabetes Association for the management of glycaemia in Type 2 diabetic patients with atherosclerotic cardiovascular disease (Davies et al., 2018). GLP‐1 receptor agonists have been shown to have a protective effect on the cardiovasculature (Ravassa, Zudaire, & Diez, 2012; Woo et al., 2013) and improve endothelial function by the activation of GLP‐1 receptors (Basalay et al., 2016; Koska et al., 2015). However, the mechanism(s) underlying vascular protection by GLP‐1 receptor agonists remain unclear (Torekov, 2018).

Currently, several studies have indicated that capillaries are vital to the regulation of organ blood flow (Biesecker et al., 2016; Fernandez‐Klett & Priller, 2015; Hall et al., 2014; Kisler, Nelson, Montagne, & Zlokovic, 2017; Peppiatt, Howarth, Mobbs, & Attwell, 2006). The blood flow in tissue can increase by 84% following nerve stimulation, with the dilatation of the capillaries preceding that of the small arteries (Hall et al., 2014). In addition, the prolonged constriction of pericytes impaired the re‐flow in capillaries even after the successful re‐opening of constricted arteries in the reperfusion period after ischaemia (Hall et al., 2014), which is the pathophysiology of a wide series of diseases including stroke and microvascular complications. Thus, relieving pericyte constriction and restoring the blood flow is a novel strategy for preventing ischaemia–reperfusion (I/R) injury (Biesecker et al., 2016; Yemisci et al., 2009). Recent studies have shown that GLP‐1 receptor agonists have a vasoactive function through regulating smooth muscle to change the diameter of blood vessels, such as mesenteric arteries, adipose tissue arterioles, and afferent arterioles (Jensen et al., 2015; Koska et al., 2015; Salheen, Panchapakesan, Pollock, & Woodman, 2015) or an area of microvascular perfusion (Chai, Zhang, Barrett, & Liu, 2014; Smits et al., 2015). While there is no data showing that GLP‐1 receptor agonists could directly affect capillaries or pericytes, the lack of such data may be related to the inability to capture in vivo measurements of the very small capillary diameters. However, as capillaries lack smooth muscle, the regulation of capillary diameter is quite different from that of other blood vessels. It is of great significance to investigate whether GLP‐1 receptor agonists can regulate capillary diameter in either physiological or pathological conditions, or both.

The retina has a great demand for oxygen supply by blood, and this tissue possesses an autoregulatory system for maintaining a constant blood flow to ensure metabolism in retinal tissue (Country, 2017). I/R injury can occur in a variety of ocular diseases, including acute attacks of glaucoma and vascular obstructive disease, and may cause irreversible damage to and functional changes in neurons (Osborne et al., 2004). The transparency of the retina is ideal for directly observing the effects on retinal vessels such as capillaries. Thus, in this study, we used a live‐vessel imaging system to evaluate the potential effect of exendin‐4, a GLP‐1 receptor agonist, on regulating capillary blood flow in the retina under physiological and pathological conditions and performed in vivo and in vitro experiments to investigate its possible mechanisms.

## 2 METHODS

### 2.1 Animals

All animal care and experimental procedures adhered to the guidelines for the Care and Use of Laboratory Animals published by the United States National Institutes of Health (revised in 2011) and were approved by EENT Hospital of Fudan University. Animal studies are reported in compliance with the ARRIVE guidelines (Kilkenny et al., 2010) and with the recommendations made by the *British Journal of Pharmacology.*

Male Sprague–Dawley rats (6 to 8 weeks old, from SLAC Laboratory Animal Co., Ltd; Shanghai, China) weighing 200 to 250 g were used in the work described here. The animals had free access to food and water under clean, temperature‐controlled conditions, in accordance with the Association for Research in Vision and Ophthalmology Statement on the Use of Animals in Ophthalmic and Vision Research. The rats were housed in groups of six to eight animals in cages containing rich bedding made of dried wood chips. Rats were selected for treatment randomly and observed without knowledge of the treatments administered.

### 2.2 Whole retina preparation

The rats were killed after anaesthetizing with 10% chloral hydrate (0.4 ml per 100 g, i.p. injection). After enucleation, the eyes were cut along the corneal limbus. The retinas were gently detached from the choroid and vitreous body and then immediately immersed in continuously oxygenated (95% O_2_, 5% CO_2_) artificial CSF (ACSF; 15‐mM glucose, 125‐mM NaCl, 3‐mM KCl, 26‐mM NaHCO_3_, 1‐mM MgCl_2_, 1.25‐mM NaH_2_PO_4_, and 2‐mM CaCl_2_). All experiments were performed at temperatures ranging from 23 to 25°C.

### 2.3 Capillary imaging

As the entire retina curled inward, it was cut into four quadrants with the vitreous side up after detachment from the eye and then fixed to the recording chamber bath installed on an upright microscope by a tungsten ring with a nylon mesh. The bath was perfused with oxygenated ACSF with or without drugs at a rate of 5 ml·min^−1^. Using infra‐red differential interference contrast (IR‐DIC) microscopy, the capillaries were visualized under a 40× water immersion objective and then captured by a video capture box (Zhongan Vision Co., Ltd; Shenzhen, China) and video recording software (Open Broadcaster Software, 22.0.2). The changes in capillary diameter and pericyte morphology were recorded during the experiments. The pixel size was 112 nm. The capillary diameters were measured at the same location marked as the middle point of the same pericyte using ImageJ software (Fuji Version, RRID:SCR_003070) (Schindelin et al., 2012). Vessel diameter was expressed as percentage of baseline value, in order to reduce unwanted sources of variation. As we used paired analysis, randomization was not needed.

To further test the effects of exendin‐4 on retinal vessels, capillaries were precontracted by noradrenaline (6 μM). After the contraction was stabilized, exendin‐4 (20 μM) was administered in the perfusing ACSF. Because the contractile response to noradrenaline varied between capillaries, only those capillaries with a contracted diameter less than 80% of the original diameter were selected for further analysis. The inhibitors used (including exendin‐9‐39, l‐NAME, 1*H*‐[1,2,4]oxadiazolo[4,3‐*a*]quinoxalin‐1‐one (ODQ), and indomethacin) were added after the addition of exendin‐4. For denuding endothelium, the retina was preincubated for 10 min with 0.3% CHAPS (McNeish, Wilson, & Martin, 2001). One researcher carried out the vascular reactivity experiments and another performed imaging and calculations of blood vessel diameters. Five rats were used in each vascular experiment. Sample size of retinal capillaries and pericytes varied and depended on the number of clear, countable pericytes contained in each image (Hall et al., 2014).

### 2.4 Experimental protocol of ischaemia–reperfusion (I/R)

Rats were anaesthetized (10% chloral hydrate, i.p.; 0.4ml per 100g) and tropicamide eye drops and oxybuprocaine hydrochloride eye drops (Santen, Japan) were applied locally to the right eye for pupil dilation and topical anaesthetization, respectively. Next, a needle was inserted into the anterior chamber and connected to a syringe pump set with physiological saline at a vertical distance to achieve increased intraocular pressure (Figure S2). Under the operation microscope, the fundus blood vessels appeared extremely thin and almost severed (Figure 4a,b). The intraocular pressure was monitored using a tonometer (TonoLab, ICare; Espoo, Finland). After the high pressure‐induced ischaemia was maintained for 1 h, the needle was removed, and refilling of the fundus vessels could be observed. The rats were placed in a warm and clean environment for fundus blood flow reperfusion for 2 h. Then, the animals were killed with an overdose of chloral hydrate. Retinal detachment was performed as described above, and the isolated retinas were used in both DIC and in vivo experiments. This model of I/R in rats reproduces aspects of I/R injury in humans (Goncalves et al., 2016), including ocular diseases such as retinal vascular occlusion, acute glaucoma and diabetic retinopathy, myocardial infarction, and cerebral stroke. At the beginning, there were 10 rats in each group. Ductal slippage during high pressure ischaemia, retinal fragmentation, and other reasons may lead to the failure of the model, and such animals were excluded from the next experiment.

For the DIC experiments, the detached retina was immediately immersed in ACSF and gassed continually with 95% O_2_; 5% CO_2_ to permit observation of the capillaries and pericytes. After the capillaries reached a stable state, exendin‐4 (final concentration, 20 μM) was added to the perfusion solution to further study its effects on retinal capillaries, under I/R conditions.

For the in vivo experiments, the retina was used to investigate changes in certain target proteins. First, exendin‐4 or vehicles were applied to rats by either subcutaneous injection (1 or 10 μg·kg^−1^), eye drops (2.5 or 25 μg·kg^−1^, at the concentration of 0.1 or 1 μg·μl^−1^), or intravitreal injection (1 or 10 μg·kg^−1^, at the concentration of 0.1 or 1 μg·μl^−1^). Immediately after the drug administration, the needle was inserted to achieve intraocular ischaemia.

### 2.5 Cell cultures

Human retinal microvascular endothelial cells (HRMECs) (cAP‐0010, Angio‐Proteomie; Boston, MA, USA) were grown and maintained in endothelial growth medium (EGM, 1001, ScienCell; Carlsbad, CA, USA) containing 10% FBS and 1% penicillin/streptomycin in 5% CO_2_ at 37°C. Human retinal pericyte cells (HRPs) (ACBRI 183, Cell Systems, Kirkland, WA, USA) were grown and maintained in complete medium (4Z0‐500, Cell Systems, Kirkland, WA, USA) with 10% FBS in 5% CO_2_ at 37°C. HRMECs were used before eight passages. HRPs were used before five passages.

### 2.6 Oxygen‐glucose deprived/reperfusion (OGD/R) model

Briefly, the HRMECs were seeded and cultured under normal conditions (21% O_2_, 5% CO_2_) at 37°C. When the HRMECs were cultured to reach 80% confluency, the original medium (EGM) was removed and replaced with glucose‐free DMEM (Gibco, Carlsbad, CA, USA). The cells were placed in hypoxic conditions (0.1% O_2_, 94.9% N_2_, and 5% CO_2_) at 37°C for 8 h. Then, the DMEM was discarded and the EGM with or without administration of exendin‐4 (1 nM), exendin‐9–39 (low dose: 10 nM; high dose: 20 nM), and l‐NAME (low dose: 50 μM; high dose: 100 μM) were added to incubate HRMECs during reperfusion in normoxic conditions (21% O_2_, 5% CO_2_) for 16 h. HRMECs maintained in EGM and under normoxic conditions were regarded as the control group.

### 2.7 NO detection

HRMECs (10 × 10^4^ cells per well) were seeded in six‐well plates. Then, the cells were collected and lysed by RIPA buffer (P0013B, Beyotime, Haimen, China) and centrifuged at 15777 x *g* for 15 min at 4°C. The supernatants were collected for assessment of intracellular NO levels by the Griess Reagent System (#G2930, Promega, Madison, USA).

### 2.8 Immunofluorescence

The whole retina was placed into a 24‐pore plate and fixed in 4% paraformaldehyde at temperatures ranging from 23 to 25°C. After 40 min, the retina was washed with PBS three times and incubated in PBS containing 2% Triton X‐100 for 1 h. Next, the tissue was blocked using PBS with 3% BSA for 1 h at room temperature, followed by incubation with primary antibodies GLP‐1R (1:200, Santa Cruz Biotechnology, Inc.; Dallas, TX, USA, Cat# sc‐66,911, RRID:AB_2110037), RECA‐1 (1:200, Cat# ab9774, RRID:AB_296613, Abcam; Cambridge, MA, USA), and NG‐2 (1:200, Cat# ab50009, RRID:AB_881569, Abcam) overnight at 4°C. The retina was then incubated with secondary goat‐anti‐mouse IgG (Alexa Fluor 488, Abcam, Cat# ab150117, RRID:AB_2688012) and goat‐anti‐rabbit IgG (Cy3, Abcam, Cat# ab6939, RRID:AB_955021) antibodies each at a concentration of 1:1,000 for 1 h. Afterward, the retina was washed three times with PBS, gently removed from the well plate in the dark, flattened on the slide with the vitreous body side up, and the fluorescent sealing liquid applied. A Leica confocal microscope (Heidelberg, Germany) was used for observations.

The immunofluorescence of cells was measured with a procedure similar to that of the retinal tissue. Briefly, cells were grown on coverslips to a density of about 80% and then washed with PBS. After fixing with 4% paraformaldehyde for 10 min followed by permeabilization with PBS containing 1% Triton X‐100 for 20 min, the coverslips were washed with PBS and then blocked using 3% BSA/PBS for 1 h. The following primary antibodies were used for staining overnight at 4°C. For HRMECs: anti‐GLP‐1R (1:200, RRID:AB_2110043, Novus; Centennial, CO, USA) and anti‐ET‐1 (1:200, Cat# ab2786, RRID:AB_303299, Abcam). For HRPs: anti‐GLP‐1R (1:200, RRID: AB_2110043, Novus; Centennial, CO, USA) and anti‐α‐SMA (1:200, Cat# ab21027, RRID:AB_1951138, Abcam). After a PBS wash, the coverslips were incubated with secondary antibodies for 1 h at room temperature, followed by staining with DAPI (1:1,000, Cat# D9542, Sigma‐Aldrich, Saint Louis, USA) for 10 min. Secondary antibodies for HRMECs: Alexa Fluor 488 donkey anti‐mouse IgG (1:1,000, Cat# ab150109, RRID:AB_2571721, Abcam) and Cy3 donkey anti‐rabbit IgG secondary antibodies (1:1,000, Cat# ab150064, RRID:AB_2734146, Abcam). Secondary antibodies for HRPs: Alexa Fluor 647 donkey anti‐goat IgG (1:1,000, Cat# ab150135, RRID:AB_2687955, Abcam) and Cy3 donkey anti‐rabbit IgG secondary antibodies (1:1,000, RRID: AB_2734146, Abcam). The immuno‐related procedures used comply with the recommendations made by the *British Journal of Pharmacology* (Alexander et al., 2018).

### 2.9 Capillary western blot

Protein expression of rat retina was detected by capillary western blot (Wes, Protein Simple; San Jose, CA, USA), which is considered as a more accurate, efficient, and sensitive measurement method than traditional western, which has been applied in many studies (Back et al., 2019; Du et al., 2018). All experimental steps were carried out according to the manufacturer's instructions. Briefly, after protein extraction and quantification of retina tissue, 1.5‐μg total protein was added to the capillary cartridges (12–230 kDa Wes Separation Module 8 × 25 capillary cartridges, Cat#SM‐W004) for each protein to be separated. Using the Wes system, the proteins were detected automatically with corresponding primary antibodies, including β‐actin (1:200, Cat#3700S, RRID:AB_2242334, Cell Signalling Technology; Denver, MA, USA), eNOS (1:50, Cat# ab199956, Abcam), p‐eNOS (1:50, Cat#9571S, RRID:AB_329837, Cell Signalling Technology), GLP‐1R (1:50, Cat# NLS1205, RRID:AB_2110043, NOVUS), Akt (1:50, Cat# 9272, RRID:AB_329827, Cell Signalling Technology), and PI3K (1:50, Cat# 60225‐1‐Ig, RRID:AB_11042594 Proteintech). The calculation and analysis of protein expression were based on the gel‐like images produced by the Compass for SW software (Version 4.0, Protein Simple). The value was expressed as “fold mean of the controls” for reducing unwanted sources of variation. The experimental details of the western blot conform to the *BJP* guidelines (Alexander et al., 2018).

### 2.10 Data and statistical analysis

The data and statistical analysis comply with the recommendations of the *British Journal of Pharmacology* on experimental design and analysis in pharmacology (Curtis et al., 2018). The results are presented as mean ± SEM. Given the past experience, each group contained at least five different subjects. The group size is the number of independent values, and the statistical analysis was done using these independent values. Outliers were included in data analysis. All vessel data were expressed as actual vessel diameters or the normalized percentage of the initial diameters before noradrenaline treatment. Following data transformation, units of a variable were determined as “normalized vessel diameter (%),” “normalized pericyte width (%),” or “fold mean of the controls,” as the Y‐axis label. Student's *t*‐tests and one‐way ANOVA with LSD or Dunnett's T3 test was used for group comparison. Paired *t*‐tests and randomized block design ANOVA with an LSD test were used for comparing vascular responses. The correlation between pericyte width and the change in vessel diameter was analysed by linear regression and calculated by Pearson correlation coefficients. Statistical significance was set as *P <.*05. Post hoc tests were conducted only if *F* achieved *P* < 0.05. IBM SPSS Statistics 22.0 (SPSS: RRID:SCR_002865) was used for the statistical analysis. Statistical analysis was undertaken only for studies where each group size was at least *n* = 5.

### 2.11 Materials

Exendin‐4, exendin‐9‐39, and noradrenaline bitartrate monohydrate were purchased from MedChemExpress (Monmouth Junction, NJ, USA). *N* ^ω^‐nitro‐l‐arginine methyl ester hydrochloride (l ‐NAME: inhibitor of endothelial NOS), indomethacin (inhibitor of COX), sodium chloride, sodium phosphate monobasic, potassium chloride, sodium bicarbonate, d‐(+)glucose, calcium chloride dehydrate, magnesium chloride hexahydrate, 1*H*‐[1,2,4]oxadiazolo[4,3‐*a*]quinoxalin‐1‐one (ODQ: inhibitor of cGMP), DMSO, 3‐[(3‐cholamidopropyl) dimethylammonio]‐1‐propanesulfonate (CHAPS), and DAPI were purchased from Sigma Aldrich (St. Louis, MO, USA). BSA was purchased from YEASEN Biotech Co., Ltd. (Shanghai, China).

### 2.12 Nomenclature of targets and ligands

Key protein targets and ligands in this article are hyperlinked to corresponding entries in http://www.guidetopharmacology.org, the common portal for data from the IUPHAR/BPS Guide to PHARMACOLOGY (Harding et al., 2018), and are permanently archived in the Concise Guide to PHARMACOLOGY 2019/20 (Alexander, Christopoulos et al., 2019; Alexander, Fabbro et al., 2019).

## 3 RESULTS

### 3.1 Expression of GLP‐1 receptors on retinal capillaries

**FIGURE 1:**
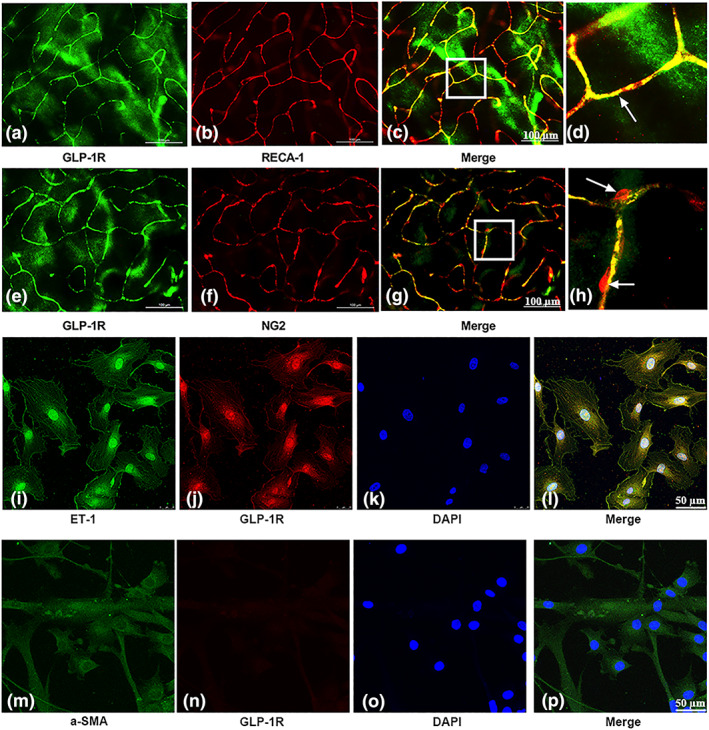
Location of GLP‐1 receptors and effects of the receptor agonist exendin‐4 on retinal capillaries. (a–h) Expression of GLP‐1 receptors (GLP‐!R) in retinal capillaries. Scale bar: 100 μm. (a, e) GLP‐1 receptors are labelled in green. (b) Endothelial cells labelled with RECA‐1 antibodies (red). (f) Pericytes labelled with NG2 antibodies (red). (c, g) Merged images. (d and h) Enlargement of the inset of (c) and (d), respectively. (i–l) Expression of GLP‐1 receptors in human retinal microvascular endothelial cells (HRMECs). (i) Endothelial cells labelled with endothelin‐1 (ET‐1) antibodies (green). (j) GLP‐1 receptors are labelled in red. (k) DAPI staining indicates the cell nucleus (blue). (l) Merged images. (m–p) Evaluation of GLP‐1 receptors expression in human retinal pericytes (HRPs). (m) Pericytes labelled with α‐SMA antibodies (green). (n) GLP‐1 receptors are labelled in red. (o) DAPI staining indicates the cell nucleus (blue). (l) merged images

Retinal patch immunofluorescence was used to assess whether the GLP‐1 receptor existed in retina microvessels. The results show that the GLP‐1 receptor was co‐expressed with the specific marker (RECA‐1) of endothelial cells in capillaries (Figure 1a–d). However, the GLP‐1 receptor was not present in NG2‐labelled pericytes (Figure 1e–h). In addition, the GLP‐1 receptor was present in the HRMECs (Figure 1i–l) but not in HRPs (Figure 1m–p). The expression of mRNA for GLP‐1 receptors in primary human retinal pericytes (HRPs) was evaluated using quantitative real‐time PCR assay. The results indicated that the expression of mRNA for GLP‐1 receptors was very low or even undetectable in HRPs, using our experimental system and technique (data not shown).

### 3.2 Effect of exendin‐4 on tone of retinal capillaries

**FIGURE 2:**
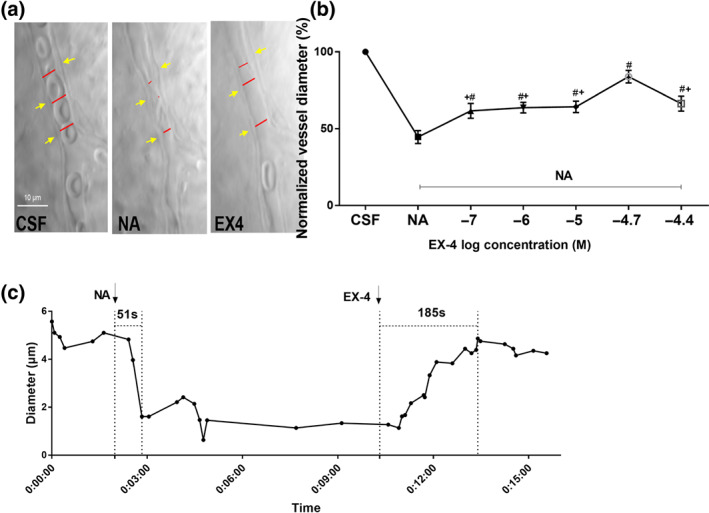
Noradrenaline‐precontracted retinal capillaries were dilated by exendin‐4. (a) Images showing changes in the rat retinal capillaries in response to 6 μM noradrenaline and 20‐μM exendin‐4. The red lines indicate the lumen diameter. The yellow arrow indicates the pericyte soma. Scale bar: 10 μm. (b) Effects of different concentrations of exendin‐4 on capillaries precontracted with noradrenaline (NA). Randomized block ANOVA followed by an LSD test (*n* = 27, 26, 19, 27, 27, 26, 19, for group of control, NA, NA + 0.1 μM exendin‐4, NA + 1 μM exendin‐4, NA + 10 μM exendin‐4, NA + 20 μM exendin‐4, NA + 40 μM exendin‐4, respectively; ^#^ *P* < .05, significantly different from noradrenaline without exendin‐4 group; ^*^ *P* < .05, significantly different from NA + 20‐μM exendin‐4 group. Log transformation was used to generate a Gaussian‐distributed data set amenable to parametric statistical analysis. (c) The time course curve showed capillaries dilated by exendin‐4. CSF, artificial CSF. NA, noradrenaline. EX‐4, exendin‐4

After administration of noradrenaline (6 μM), the capillaries were strongly contracted (Figure 2a,b). The application of exendin‐4 dilated the pre‐constricted retinal capillaries (NA + Exendin‐4; 0, 0.1, 1, 10, 20, 40 μM; Figure 2b). After the administration of exendin‐4 at 20 μM, the diameter of pre‐constricted capillaries was clearly increased (contraction reversed) towards baseline values. At 20 μM exendin‐4, the reversal was significantly greater than at 10 μM or 40 μM and thus, 20 μM exendin‐4 was chosen as the concentration to be used in in the subsequent experiments.

Figure 2c shows the changes in the diameter of a single retinal capillary region after the successive addition of noradrenaline and exendin‐4. After perfusion with noradrenaline for 51 s, the capillary rapidly narrowed, contracting from 5.1 to 1.6 μm (~9.3% of the original diameter). Exendin‐4 (20 μM) was added as the contraction stabilized during the continuous noradrenaline perfusion (diameter was 1.336 μm). After perfusion of exendin‐4 for 185 s, the retinal capillary diameter gradually increased to 4.9 μm, close to the initial diameter, and maintained this size thereafter.

### 3.3 Mechanism of exendin‐4‐induced relaxation of retinal capillaries

**FIGURE 3:**
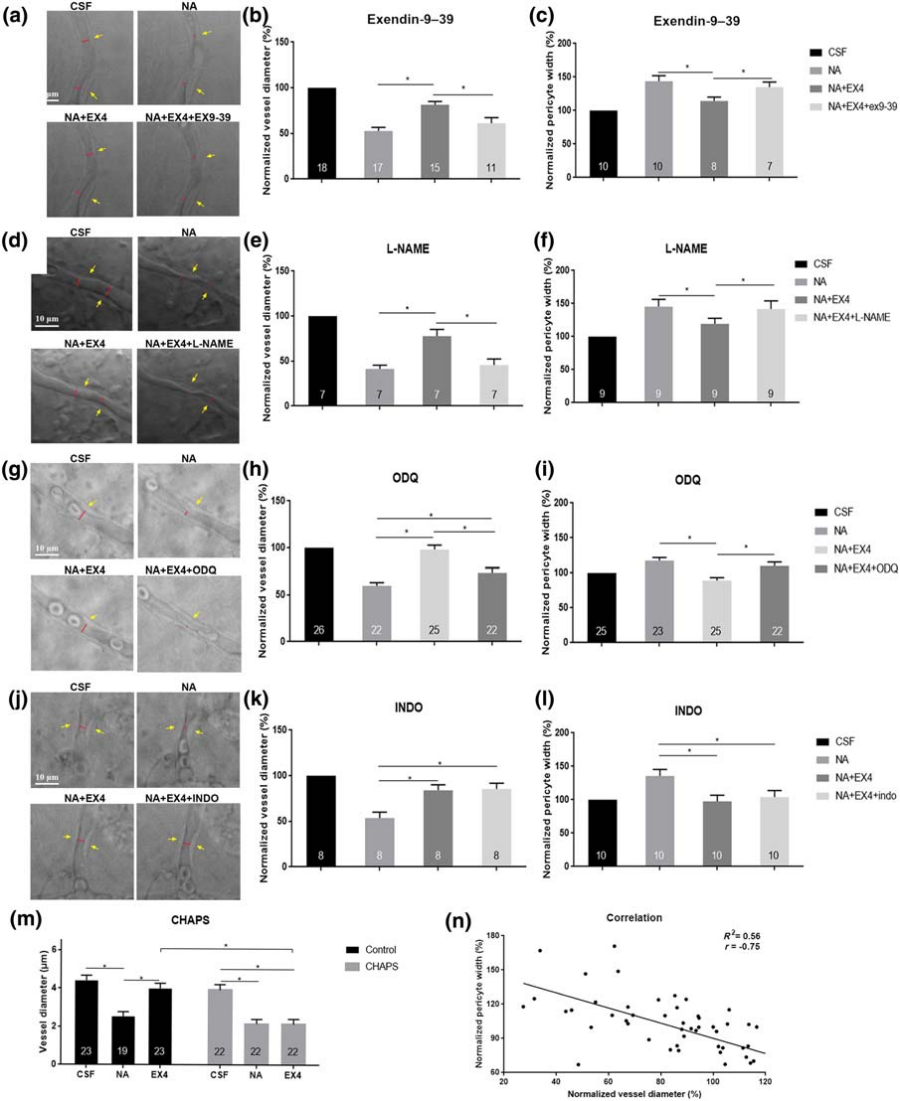
Signalling pathways of exendin‐4‐induced vasodilation. (a, d, g, and j) Images depict the effect of inhibitors on exendin‐4‐dilated capillaries. (b, e, h, and k) Percentage change in retinal capillary diameter. (c, f, i, and l) Percentage change in pericyte width in the corresponding area. (a–c) Exendin‐9–39‐attenuated, exendin‐4‐induced vasodilation. (d–f) l‐NAME inhibits exendin‐4‐induced vasodilation. (g–i) ODQ‐reduced exendin‐4‐induced relaxation of capillaries. (j–l) Indomethacin does not inhibit exendin‐4‐induced vasodilation. The red lines indicate the lumen diameter. The yellow arrow indicates the pericyte soma. Scale bar: 10 μm. Randomized block ANOVA followed by an LSD test (*N* was indicated in each column of the graph). ^*^ *P* < 0.05, significantly different as indicated. CSF, artificial CSF; NA, noradrenaline; EX‐4, exendin‐4; EX‐9–39, exendin‐9–39; INDO, indomethacin. (m) CHAPS abolishes exendin‐4‐induced vasodilation. (n) Linear regression analysis for correlation between capillary diameter and change in pericyte width (*n* = 69 pairs)

To first evaluate whether exendin‐4 causes capillary dilation through binding to the GLP‐1 receptor, exendin‐9–39 (a GLP‐1 receptor antagonist) was added to perfusate that included noradrenaline and exendin‐4 (Figure 3a–c). The results reveal that exendin‐9–39 significantly attenuated the vasorelaxation effect of exendin‐4 on pre‐constricted capillaries (Figure 3b). Next, we evaluated whether the eNOS/NO/cGMP pathway was involved in the vasodilator effect of exendin‐4 (Figure 3d–f). The administration of l‐NAME (an inhibitor of NOS) inhibited the capillary relaxation induced by exendin‐4 (Figure 3e) and pericytes relaxation (Figure 3f). ODQ, which blocks GC (Figure 3g–i), also significantly reduced the exendin‐4‐induced relaxation of capillaries and changes in the width of the pericytes. However, the capillary dilation and pericyte flattening caused by exendin‐4 were not affected by blocking COX with indomethacin (*P* = 0.82, Figure 3j–l).

To investigate whether the regulation of exendin‐4 on blood vessels depends on endothelial cells, we carried out chemical removal of endothelium with 0.3% CHAPS. Pretreatment with CHAPS had no significant effect on the diameter of non‐precontracted capillaries or on the diameter of noradrenaline‐precontracted capillaries. But CHAPS preincubation almost abolished the vasodilator effects of exendin‐4 (Figure 3m).

To further evaluate the relationship between pericyte and capillary regulation, linear correlation analysis was conducted between the normalized width of the pericyte cell body and the normalized diameter of the capillary in the corresponding area after administration of noradrenaline or noradrenaline with exendin‐4. Figure 3n shows the significant, negative correlation between the two indices.

### 3.4 Effect of exendin‐4 on the tone of retinal capillaries under ischaemic–reperfusion conditions

**FIGURE 4:**
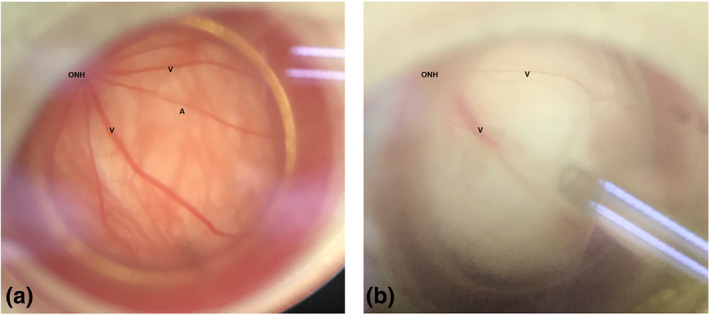
A model of ocular ischaemia–reperfusion induced by high perfusion pressure in the anterior chamber. (a, b) Fundus photographs of retinal vascular perfusion before (a) and during (b) ischaemia injury. During ischaemia injury, the retinal arteries were almost invisible

**FIGURE 5:**
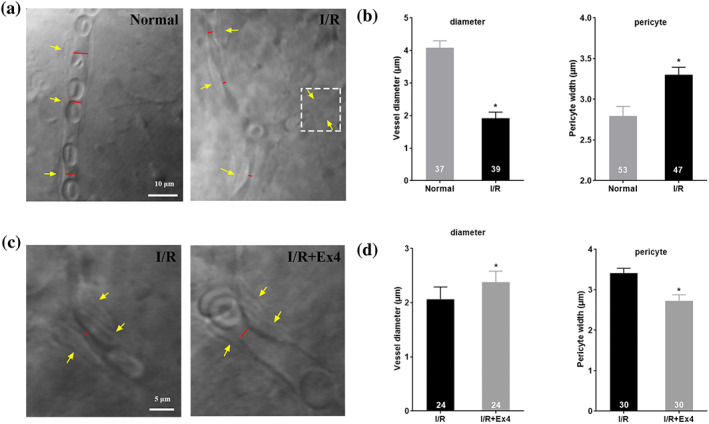
Exendin‐4 restores microvascular patency of capillary negatively affected by I/R injury. (a) Representative images of capillaries after I/R. I/R‐induced pericyte contraction narrowed the lumen diameter and even completely blocked the capillary (in white dotted box). (b) Changes in diameter and pericyte soma width after I/R. (c and d) perfusion of exendin‐4 relieved the contracted pericyte and dilated narrow, injured capillaries (increasing the capillary diameter). The red lines indicate the lumen diameter. The yellow arrow indicates the pericyte soma. Student's *t*‐test and paired *t*‐tests were performed. *N* is indicated in each column of the graph. In (b), ^*^ *P* < .05, significantly different from Normal (b); in (d), ^*^ *P* < .05, significantly different from I/R. EX‐4, exendin‐4. I/R, ischaemia–reperfusion

Under ischaemic conditions, the retinal arteries appeared to be almost blocked at a perfusion pressure of up to 110 mmHg (Figure 4a,b). In DIC systems, the retinal capillaries that had been exposed to I/R injury were markedly narrow and sometimes even blocked by constricted pericytes (Figure 5a). Figure 5b shows that in the I/R group, capillary diameter was significantly reduced and pericytes were contracted, when compared with normal retinal capillaries.

Further study revealed that, after exendin‐4 was added to ACSF for continuous perfusion, the capillary diameter gradually increased, with the gradual dilation of pericytes, in retina that had been exposed to I/R procedures (Figure 5c,d).

### 3.5 Effect of exendin‐4 on retina under ischaemic–reperfusion conditions

To further study the possible pathway of exendin‐4 under retinal ischaemic–reperfusion conditions, the rats were pretreated with exendin‐4, different methods of administration, prior to inducing retinal I/R injury. Compared with the control group, the expression of mRNA for GLP‐1 receptors was significantly decreased in the I/R model group. However, the exogenous administration of exendin‐4 did not increase the transcription level of GLP‐1 receptors (Figure S1A). Levels of mRNA for eNOS in the I/R group were significantly lower than that in the control group and no difference was found during the I/R injury, with or without exendin‐4 treatment (Figure S1B).

**FIGURE 6:**
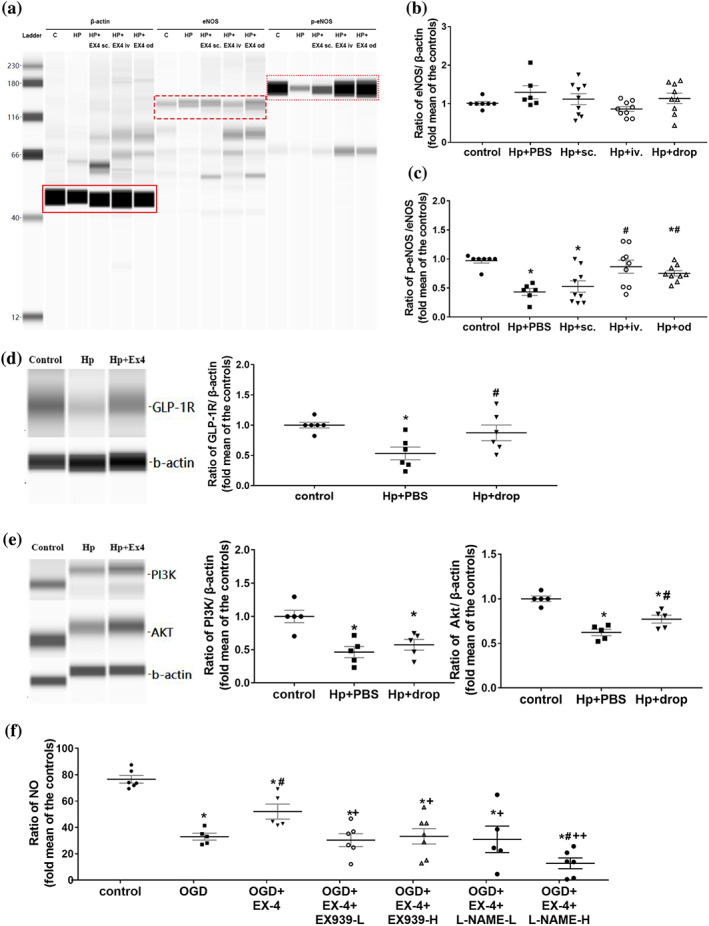
Effect of the administration of exendin‐4 on eNOS expression. (a) Capillary western blot of total eNOS and phosphorylated eNOS expression in the rat retina. The red box indicates the target protein. (b and c) Fold change in the expression of total eNOS (b) and phosphorylated eNOS (c) in the rat retina (*n* = 7 in control group; *n* = 6 in Hp + PBS group; *n* = 9 in other groups). (d and e) Capillary western blot and fold change in the expression of GLP‐1 receptors (GLP‐1R) (d), PI3K, and Akt (e) in the rat retina. ^*^ *P* < .05, significantly different from CSF group; ^#^ *P* < .05significantly different from noradrenaline without exendin‐4 group. (f) NO content in human retinal microvascular endothelial cells (*n* = 6, 5, 5, 6, 7, 5, and 6 for group of control, OGD, OGD + Ex‐4, OGD + Ex‐4 + Ex‐9–39‐L, OGD + Ex‐4 + Ex‐9–39‐H, OGD + Ex‐4 + l‐NAME‐L, and OGD + Ex‐4 + l‐NAME‐H, respectively). One‐way ANOVA with LSD or Dunnett's T3 test were performed. C, control group; HP, high pressure injury group; EX‐4, exendin‐4; s.c., subcutaneous injection of exendin‐4; i.v., intravitreal injection of exendin‐4; od, eye drops of exendin‐4; OGD, oxygen glucose deprivation model; EX‐9–39‐L, low concentration of exendin‐9‐39 (10 nM); EX‐9–39‐H, high concentration of exendin‐9‐39 (20 nM); l‐NAME‐l, low concentration of l‐NAME (50 μM); l‐NAME‐H, high concentration of l‐NAME (100 μM)

The expression of total eNOS protein showed no difference among all groups (Figure 6a,b). However, the phosphorylation level of eNOS protein in the I/R group was significantly lower than that of the control group (Figure 6a,c). After administering exendin‐4 by intravitreal injection or in eye drops, the phosphorylated level of eNOS level was normalized in retina with I/R injury. Administration of exendin‐4 in eye drops almost reversed the decrease in translation of GLP‐1 receptors caused by I/R (Figure 6d). Compared with the control group, PI3K expression was significantly reduced after I/R injury group and treatment with exendin‐4 showed a trend towards reversing that reduction. However exendin‐4 did reverse the decreased level of Akt caused by I/R injury (Figure 6e).

### 3.6 Effect of exendin‐4 on blood glucose under ischaemic–reperfusion conditions

As exendin‐4 affects blood glucose in patients with diabetes, we investigated possible changes in the blood glucose level after the administration of exendin‐4 through different routes. No significant difference was found among the control group, low and high dosage subcutaneous injection groups, low and high dosage intravitreal injection groups, and low and high dosage eye drop groups (Figure S1C).

### 3.7 Exendin‐4 ameliorated the reduction of NO levels induced by OGD/R in HRMECs

To further investigate the molecular mechanism of exendin‐4 effect, we examined the intracellular NO levels of HRMECs induced by OGD/R. As shown in Figure 6f, the average NO levels of HRMECs significantly decreased after OGD/R insult. Administration of exendin‐4 during reperfusion partly protected against this OGD/R‐induced reduction of NO. The addition of exendin‐9–39 (GLP‐1 receptor antagonist) or l‐NAME (eNOS inhibitor) eliminated the effect of exendin‐4 on NO levels. These results confirmed that treatment with exendin‐4 ameliorated the reduction of NO induced by OGD/R, through a GLP‐1 receptor/eNOS pathway.

## 4 DISCUSSION

The present study demonstrated that GLP‐1 receptors were present on the retinal capillary endothelium and that exendin‐4 could effectively regulate the diameter of retinal capillary through the GLP‐1 receptor‐PI3K/Akt‐eNOS/NO‐cGMP pathway. Moreover, this is the first time that exendin‐4 has been shown to relieve the narrowing of capillaries induced by I/R injury. In addition, the topical administration of exendin‐4 was able improve retinal vascular endothelial function under I/R conditions.

Our results have shown that GLP‐1 receptor agonists have a direct dilator effect on the capillary tone. IR‐DIC microscopy with continuously oxygenated ACSF was used to maintain blood vessel vitality for the direct, real‐time observation of the changes in living capillaries, as has been performed previously for microvascular observations (Hall et al., 2014; Peppiatt et al., 2006; Yemisci et al., 2009; Zong et al., 2018). In addition, the isolated retina was selected as an ideal object for vascular observation due to its transparent characteristics and tissue integrity. Moreover, compared with in vivo vascular regulation by systemic delivery, the regulation by perfusion of exendin‐4 over isolated retinal capillaries under the IR‐DIC system could avoid changes in the systemic metabolism of insulin or other vasoactive substances.

In the whole‐mounted retina of normal rats, GLP‐1 receptors were detected in retinal capillary endothelial cells but not in pericytes. In addition, GLP‐1 receptors were expressed in cultured HRMECs but not in HRPs. Some studies have shown GLP‐1 receptors in the vascular endothelial cells of tissues such as that of the heart (Ban et al., 2008) and umbilical veins (Dai, Mehta, & Chen, 2013). However, regarding the endothelial cells in retina, a previous study using immunohistochemistry of tissue sections found no GLP‐1 receptors located in human retinal vasculature (Hebsgaard et al., 2018). The conflicting results may be due to our use of whole‐patch retina rather than tissue sections for immunohistochemistry. The retina vasculature runs within multiple layers of abundant nerve cells, which might cause difficulty in identifying the proper cross‐section containing blood vessels on the vertical surface of the retina when using tissue sections. In addition, the microvasculature could be much harder to distinguish, by immunohistochemistry, using tissue sections.

As the GLP‐1 receptors were expressed on retinal capillary endothelial cells, we further investigated the vasodilator pathways) involved. In particular, pericytes can response to NO and arachidonic acid metabolites, such as PGs, to induce capillary relaxation (Hall et al., 2014). In the present study, inhibitors of eNOS or cGMP blocked dilation by exendin‐4. This observation is in line with previous reports that the exendin‐4‐mediated vasorelaxation on human arterioles (Koska et al., 2015) and rat mesenteric arteries (Salheen et al., 2015) was dependent on NO. However, the results of a human clinical study are in contrast with our findings (Smits et al., 2015). This study had found that exendin‐4‐induced vasorelaxation could not be abolished when NOS was inhibited. The differences may be explained by the different methods used to administer exendin‐4. The systemic administration used in the previous clinical study may have caused the vessel to relax through both direct and indirect means due to the effects of systemic metabolites and catabolic enzymes. Additionally, in our study, the COX inhibitor, indomethacin did not affect the vasodilation caused by exendin‐4. In capillaries, COX can metabolize arachidonic acid to produce PGE_2_, which could activate EP_4_ receptors on pericytes and cause pericyte relaxation (Kisler et al., 2017). Our results showed that the COX pathway did not participate in the exendin‐4‐induced relaxation of retinal capillaries, which was consistent with studies of exendin‐4 on isolated mesenteric arteries and the aorta (Green et al., 2008; Jensen et al., 2015).

Capillaries are considered to be controlled by pericytes or precapillary arterioles (Fernandez‐Klett & Priller, 2015; Kisler, Nelson, Rege, et al., 2017). In our study, we observed that the constriction or dilatation of the capillary was accompanied by corresponding changes in the pericyte. In addition, correlation analysis indicates a significant correlation between changes in the capillary diameter and pericyte width. These data would support the possibility of the dilatation of capillaries by exendin‐4 through the relaxation of pericytes. Additionally, after denuding endothelium with CHAPS, exendin‐4 did not dilate the pre‐constricted retinal capillaries, which demonstrates that the relaxation of exendin‐4 on capillaries was dependent on endothelial cells. It would also indicate that the capillary relaxation induced by exendin‐4 was dependent on endothelial function and involved pericytes. Further investigation is needed, however, to determine the exact mechanism of the interactions of the endothelial cells with pericytes.

I/R injury models were designed to further explore whether exendin‐4 could also regulate retinal capillaries under disease conditions. The present study showed, using DIC systems, that the pericyte constricted and even blocked the capillary flow under reperfusion conditions after ischaemia. The model mimicked pathological injury in clinical settings, such as a grand attack of acute angle‐closure glaucoma, in which the patient intraocular pressure often rises sharply, up to 70 mmHg. Similar to our findings, even if the intraocular pressure is reduced and artery blood supply is restored, damage still occurs in the peripapillary retinal vessels (Wang, Jiang, Kong, Yu, & Sun, 2017). Previous laboratory research has confirmed that pericyte control of capillaries plays a critical role in ischaemia and that the capillary constriction or even “no‐reflow” induced by pericyte outlasts the period of ischaemia. (Hall et al., 2014; Yemisci et al., 2009), which also corresponds with our results. A broken balance of vasoactive molecules, overload of calcium fluxes in pericytes (Hamilton, 2010; Peppiatt et al., 2006), and oxidative‐nitrative stress induced by I/R injury could be the possible reason for this prolonged effect. Interestingly, after the injured retina capillaries were superfused with exendin‐4, capillary constriction was clearly relieved. Restoring capillary patency is of vital significance for tissue survival (Yemisci et al., 2009). To our knowledge, this is the first report that exendin‐4 could relieve the narrowing of the capillaries following I/R injury. This may provide a potential therapy to treat pathological events occurring in the early course of I/R injury.

To further investigate whether exendin‐4 also had effects in vivo in addition to the most likely pathway, drugs were administered to rats through different routes before the ischaemia. First, we demonstrated that the transcription and translation levels of GLP‐1 receptors was significantly decreased in the I/R model. A previous study found that expression of GLP‐1 receptors could be detected in normal human eyes but not in eyes with advanced diabetic retinopathy (Hebsgaard et al., 2018). This finding suggests that GLP‐1 receptors might be decreased in conditions involving microvascular damage, which may explain the decrease of these receptors following I/R in the present study. As the exogenous administration of exendin‐4 did not increase the transcription level of GLP‐1 receptors but increased their translation, we hypothesize that the agonist could have enhanced the number of activated receptors and thus improved the signalling function of the GLP‐1/ GLP‐1 receptor pathway. The results indicate that exendin‐4 can improve the endothelial dysfunction induced by I/R injury.

In our study, the constriction or occlusion of retinal capillaries induced by I/R injury could be ameliorated by exendin‐4. Improvement of endothelial function could underlie this finding. NO, synthesized by eNOS, is a key factor in maintaining the balance of vascular tone. It is also considered that the activation of eNOS plays a protective role on blood vessels and may be damaged under I/R conditions (Brunner et al., 2003). Our present study shows that the level of phosphorylated eNOS decreased due to I/R injury but was significantly increased by exendin‐4 administration, which was consistent with the previous studies of GLP‐1 receptor agonists on other organs such as kidney and heart after I/R injury (Abdel‐Latif, Heeba, Taye, & Khalifa, 2018; Baba et al., 2017; Chang et al., 2015; Chen et al., 2017). To further investigate whether the exendin‐4 improved the endothelial dysfunction during I/R injury through increasing NO production, the intracellular NO levels were evaluated in cultured primary HRMECs. In our present study, the OGD/R condition could reduce NO release from HRMECs and exendin‐4 could partly reverse this change. However, these effects of exendin‐4 were eliminated by blocking the GLP‐1 receptors with its antagonist (exendin‐9–39) or by inhibiting eNOS with l‐NAME. These findings indicated that the beneficial actions of exendin‐4 on endothelial cells did involve GLP‐1 receptors. In several diseases such as diabetes and hypertension, the uncoupling of eNOS characterizes the endothelial dysfunction, as it decreases production of NO and increases the generation of superoxide (Forstermann & Munzel, 2006). The decreased NO levels during OGD/R could partly be due to an exacerbation of eNOS uncoupling.

In addition, this study explored different modes of drug delivery through subcutaneous injection, intravitreal injection and eye drops. Among these, eye drops as a non‐invasive and convenient topical administration method, was able to improve the phosphorylation level of eNOS that had been decreased by I/R injury. Earlier work had shown that eye drops containing exendin‐4 were effective at preventing retinal neurodegenerative changes (Hernandez et al., 2016), and our findings would confirm the efficacy of local administration of this GLP‐1 receptor agonist. In our study, none of the three different routes of administration resulted in systemic glycaemic changes. This showed that the vascular protective effect of exendin‐4 was independent of any effects on blood glucose levels or insulin levels. In addition, no obvious ocular surface damage, such as conjunctival hyperaemia and corneal injury, was observed after applying the eye drops. This opens the possibility of developing eye drops as a safe and effective therapy in ocular conditions following I/R injury, such as acute glaucoma and retinal artery occlusion.

This study also has certain limitations. The signalling pathways related to the regulation of vasodilation and contraction include not only the pathways involving eNOS/NO and COX but also several other mediators including ET‐1 and brain natriuretic peptide, which we did not assess in our study. Therefore, it is possible that exendin‐4 may regulate capillaries in other pathways in addition to that we have identified, the PI3K/Akt‐eNOS/NO‐cGMP pathway. Furthermore, the interaction between endothelial cells and pericytes requires further investigation. We used an acute rather than a chronic injury model, as the duration of the ischaemia was only 1 h. Therefore, an experiment with longer term ischaemic damage may produce a varied response to exendin‐4. Finally, cell experiments should be used for a more comprehensive mechanistic study, and a corresponding in vitro I/R model is needed. However, in vivo and in vitro cell models of I/R injury may not be uniform. Nevertheless, further studies are needed to elucidate additional pathway details and should incorporate additional experimental groups.

In conclusion, GLP‐1 receptor agonists, such as exendin‐4, could effectively regulate the diameter of retinal capillaries through a GLP‐1 receptor‐PI3K/Akt‐eNOS/NO‐cGMP pathway under physiological conditions and after I/R injury. In addition, eye drops containing exendin‐4 may have a protective effect on retinal endothelial cells in vivo. Thus, through restoring capillary patency, exendin‐4 may be an effective treatment for improving tissue perfusion in I/R‐related diseases.

## CONFLICT OF INTEREST

The authors declare no conflicts of interest.

## AUTHOR CONTRIBUTIONS

R.Z. and H.X. performed and analysed the experiments. R.Z. wrote the first draft of the manuscript. X.S. participated in the analysis of the experiments. J.W. revised the manuscript. F.H. participated in the experiments related to qPCR, cell culture, and immunofluorescence. X.K. contributed to the experimental design and revised the manuscript.

## DECLARATION OF TRANSPARENCY AND SCIENTIFIC RIGOUR

This Declaration acknowledges that this paper adheres to the principles for transparent reporting and scientific rigour of preclinical research as stated in the BJP guidelines for Design & Analysis, Immunoblotting and Immunochemistry, and Animal Experimentation, and as recommended by funding agencies, publishers and other organisations engaged with supporting research.

## Supporting information

Supporting Information
