# Supplementary material for: Exendin‐4, a GLP‐1 receptor agonist regulates retinal capillary tone and restores microvascular patency after ischaemia–reperfusion injury
Source: Br J Pharmacol. 2020 May 27;177(15):3389–402. doi: 10.1111/bph.15059 (PMC7348095; doi:10.1111/bph.15059)
Supplement: Supplementary file 1 — Supporting Information [file BPH-177-3389-s001.pdf]

Supplementary figure 1

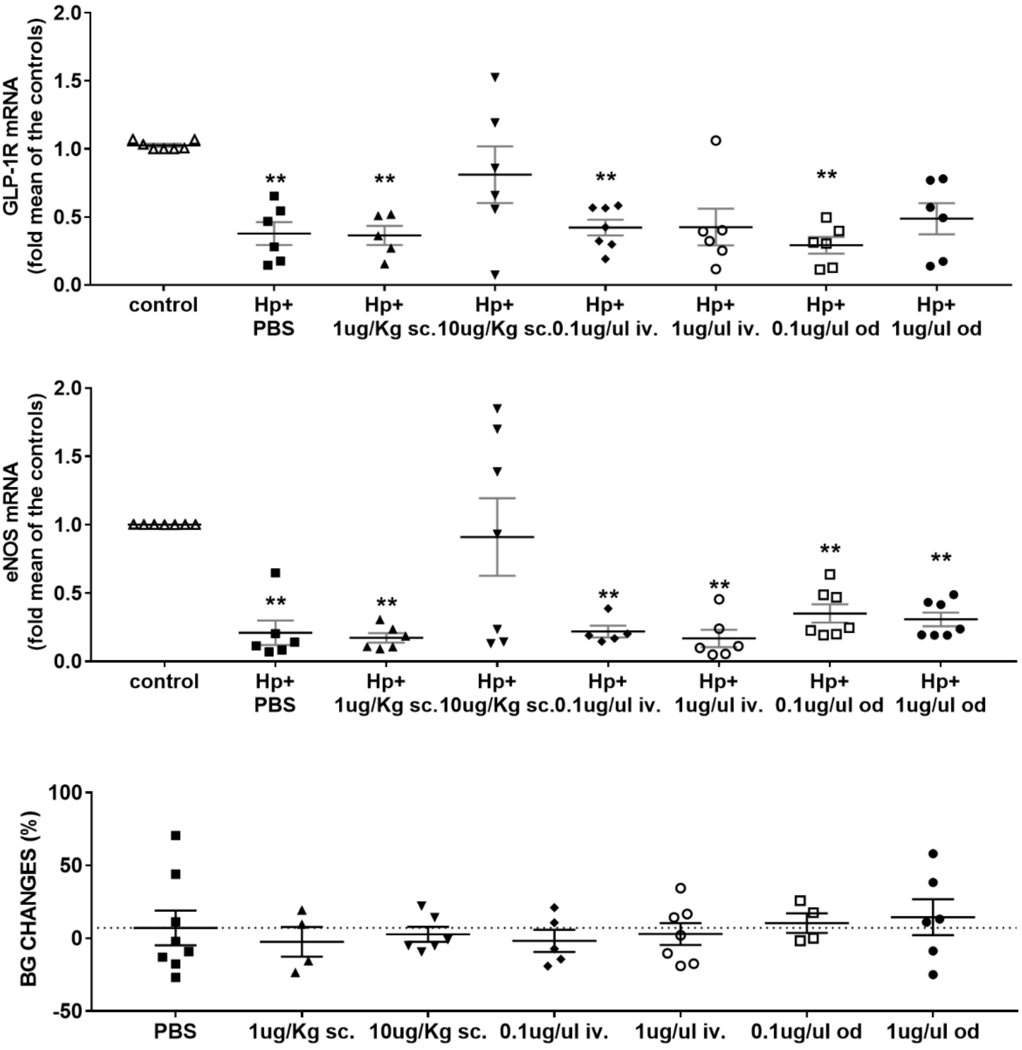

Effect of the administration of exendin-4 on the mRNA of GLP-1R (A) and eNOS (B), and the blood glucose level (C). HP, high pressure injury group. EX-4, exendin-4. sc, subcutaneous injection of exendin-4. iv, intravitreal injection of exendin-4. od, eye drops of exendin-4. A, one-way ANOVA with LSD or Dunnett’s T3 test was performed (n = 7 in control group and Hp+0.1μg/μl iv. group; n = 6 in Hp+ PBS group, Hp+10μg/Kg sc. group, Hp+1μg/μl iv. group, Hp+0.1μg/μl od group, and Hp+1μg/μl od group; and n = 5 in Hp+1μg/Kg sc. group). B, one-way ANOVA with LSD or Dunnett’s T3 test was performed (n = 7 in control group, Hp+10μg/Kg sc. group, Hp+0.1μg/μl od group, and Hp+1μg/μl od group; n = 6 in Hp+ PBS group, Hp+1μg/Kg sc. group, and Hp+1μg/μl iv. group; and n = 5 in Hp+0.1μg/μl iv. group). C, the dotted line was at 7.14% which was the mean value in control group (PBS group). Student′s t-test was performed (n = 8 in PBS group; n = 7 in 1μg/μl iv. group; n = 6 in 10μg/Kg sc. group and 1μg/μl od group; n = 5 in 0.1μg/μl iv. group; and n

= 4 in 1µg/Kg sc. group and 0.1µg/µl od group). * $p < 0.05$, versus CSF group; # $p < 0.05$, versus PBS group.

Supplementary figure 2

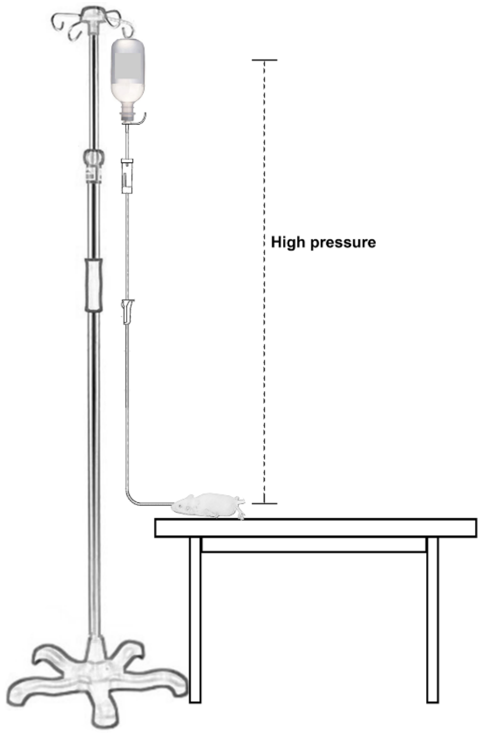

A schematic diagram of the ischemia-reperfusion model.

Supplementary method 1

Quantitative PCR

Total RNA of retina or HRPCs was extracted using the RNeasy Mini Kit (QIAGEN, Netherlands) and quantified with a spectrophotomer (NanoDrop, Thermo Scientific; Waltham, MA, USA). Complementary DNA (cDNA) was generated from total RNA through reverse transcription (TAKARA, Japan). Quantitative real-time (qRT)-PCR were performed using specific primers (Shengong, Shanghai, China) for eNOS (CACAGGCATCACCAGGAAGAAGAC and TTCACACGCTTCGCCATCACC, forward and reverse, respectively) and 4 pairs of GLP-1R primers (supplementary Table 1) with a fluorescence quantitative kit (Applied Biosystems; Foster City, CA, USA). β-actin was used as a housekeeping gene.

Supplementary Table 1 Primers for RT-PCR assay

Primer	Forward (5’→3’)	Reverse (3’→5’)
h-GLP1R-1	gaccttcgatgaatacgcctg	tcctcgcactccgacaagt
h-GLP1R-2	gtcaagtacctctatgaggacgagg	atgagtgtcagcgtggacttg
h-GLP1R-3	tggcggccaattactactg	gagccagtagttcatgttgga
h-GLP1R-4	agtccaagcgaggggaaaga	gaggcgataaccagagcagag

Supplementary method 2

Measurement of blood glucose concentration

In the I/R experiments, blood glucose levels were examined prior to exendin-4 or vehicle administration and after ischemia (ACCU-CHEK Active, Roche; Ireland).
